# Risk of Dumping Syndrome after Sleeve Gastrectomy and Roux-en-Y Gastric Bypass: Early Results of a Multicentre Prospective Study

**DOI:** 10.1155/2016/2570237

**Published:** 2016-05-08

**Authors:** M. Ramadan, M. Loureiro, K. Laughlan, R. Caiazzo, A. Iannelli, L. Brunaud, S. Czernichow, M. Nedelcu, D. Nocca

**Affiliations:** ^1^CHU de Montpellier, 80 avenue Augustin Fliche, 34090 Montpellier, France; ^2^Departamento de Biotecnologia, Universidade Positivo, Rua Angelo Bom 315, Casa 1, 81210340 Curitiba, PR, Brazil; ^3^Université Montpellier 1, Montpellier, France; ^4^Department of Surgery, Torbay Hospital, Newton Road, Torquay, Devon TQ2 7AA, UK; ^5^General Surgery, CHRU Lille, 2 avenue Oscar Lambret, 59000 Lille, France; ^6^CHU Nice, 5 rue Pierre Dévoluy, 06000 Nice, France; ^7^CHU de Nancy, 1 rue Joseph Cugnot, Nancy, France; ^8^Hôpital Ambroise-Paré, 9 avenue Charles de Gaulle, Boulogne-Billancourt, 92100 Paris, France

## Abstract

*Background*. Bariatric surgery is an important field of surgery. An important complication of bariatric surgery is dumping syndrome (DS).* Aims*. To evaluate the incidence of DS in patients undergoing bariatric surgery.* Methods*. 541 patients included from 5 nutrition and bariatric centers in France underwent either LSG or LRYGB. They were evaluated at 1 month (M1) and 6 months (M6) postoperatively by an interview and completion of a dumping syndrome questionnaire.* Results*. 268 patients underwent LSG (Group A) and 273 underwent LRYGB. From the LRYGB patients 229 had mechanical gastrojejunoanal anastomosis with 30 mm linear stapler (Group B) and 44 had manual (hand sewn) 15 mm gastrojejunal anastomosis (Group C). Overall incidence of DS was 8.5% at M1 and M6. In LSG group (Group A), only 4 patients (1.49%) reported episodes of DS at M1 and 3 (1.12%) at M6. In Group B, 41 patients (17.90%) reported episodes of DS at M1 and 43 (18.78%) at M6. Group C experienced one case (2.27%) of DS at M1 and none (0%) at M6.* Conclusions*. Patients undergoing LRYGB, especially with larger gastrojejunal anastomosis, are more prone to developing DS following surgery than patients undergoing LSG or LRYGB with calibrated manual anastomosis.

## 1. Introduction

DS is a known complication of patients following gastrectomy, although it is frequently underdiagnosed. The term “Dumping” itself was tailored by Wyllys et al. in 1920 [1]. The emergence of bariatric surgery as an important field of digestive and metabolic surgery has regained interest in the evaluation of DS.

DS includes gastrointestinal (GI) and vasomotor symptoms that occur following ingestion of a meal. Symptoms following early DS (DS occurring 15 minutes to 1 hour after a meal ingestion) are mainly GI and they are caused by osmotically driven fluid shifts from the blood to the lumen. Symptoms of late DS (DS occurring 1 hour to 3 hours following a meal) are mainly vasomotor. They are caused by reactive hypoglycemia that is induced by a surge in insulin secretion that overcompensates for the glucose load delivered to the portal circulation [2].

Early DS GI symptoms include diarrhea, nausea, epigastric fullness, borborygmus, and abdominal cramps. Late DS symptoms may include vasomotor symptoms such as sweating, decreased consciousness, shakiness, hunger, and difficulty to concentrate.

DS incidence and severity have been correlated to the type of gastrectomy performed [3], and currently it has been proven that DS occurs mainly after LRYGB operation, although the symptoms tend to improve over time [4, 5].

In our study, we aimed to evaluate the real incidence of DS among patients undergoing bariatric surgery, and particularly among patients who underwent LSG and LRYGB with different types of gastrojejunal anastomosis.

## 2. Methods

### 2.1. Study Design

Data was collected from five bariatric centers (CHRU Montpellier, CHU Lille, CHU Nancy, CHU Nice, and CHU Ambroise Paré Boulogne-Billancourt). From the period between November 2012 and May 2013, 541 patients, 409 (75.6%) females and 132 (24.4%) males, of mean age 41 years (16–70) and mean BMI 45 kg/m^2^ (31.6–83) underwent either LSG or LRYGB.

### 2.2. Operation Technique

The surgical procedures were either primary bariatric surgery for 457 patients (84.4%), or reoperations following another bariatric surgery in the remaining 84 patients (15.6%). Among those patients undergoing a second procedure 82 (97.6%) had a primary adjustable gastric band, 1 (1.1%) had a previous minigastric bypass, and 1 (1.1%) had a vertical banded gastroplasty. Procedures were all done under general anesthesia and were either completed laparoscopically for 513 patients (94.8%) or robotically assisted in 28 patients undergoing LRYGB (5.2%), involving an average of 4–6 trocars.

The LRYGB procedure was performed using a standard antecolic-antegastric Roux-en-Y construction with a 10–20 cc gastric pouch and a 150 cm Roux limb. The gastrojejunal anastomosis was performed with a 30 mm diameter using a linear mechanical stapling device (229 patients in CHU Lille, CHU Nancy, and CHU Ambroise Paré, Paris) or with a hand sewn technique of 15 mm diameter (44 patients in CHU Nice).

The LSG procedure included creating a 150 cc longitudinal gastric pouch calibrated using a 37–39-French Foucher tube or the MidSleeve® tube, after removing the gastric fundus, using linear mechanical staplers and starting 6 cm away from the pylorus until reaching the left crus.

### 2.3. Sigstad Scale and Patient Monitoring

The diagnosis of dumping syndrome was based on the Sigstad scoring system (Table 1), where a score of 7 and above was considered positive [6]. Patients were evaluated postoperatively at M1 and M6 during a thorough interview which included filling out the Sigstad questionnaire after patient's consent. In our study we also monitored the complication rates of these different procedures according to current norms and guidelines.

### 2.4. Glucose Tolerance Test and Screening for Orthostatic Hypotension

If the presenting symptoms were not clearly related to DS, especially in cases of malaise, patients underwent a 3-hour oral glucose tolerance test (OGTT) combined with screening for orthostatic hypotension to confirm the diagnosis. The test consisted of the following steps.

Informing the patient and explaining the steps of OGTT

- patients were instructed to remain fasting 12 hours before OGTT;
- patients were instructed to remain fasting and prohibited from smoking during the test;
- patients were asked to remain in the semiseated (Semi-Fowler's) position or lying down during the test;
- a catheter was inserted in a vein of the forearm during the test if possible (in certain cases several separate blood withdrawals were done).

*Procedure*. Fasting blood glucose level was measured before administering oral glucose. Afterwards, patients received an oral bolus of 75 g of glucose in 250 to 300 mL of water to be taken in less than 5 minutes. Blood glucose level, insulinemia, and C Peptide levels at 0, 60, 120, and 180 minutes were measured.

OGTT was not performed during periods of physiological stress (infection, surgery, pregnancy, myocardial infarction, etc.) or within a 3-month interval.

Medications that may interfere with glucose metabolism were also taken into consideration: loop diuretics, thiazides, corticosteroids, estrogens, progestogens, danazol, *β*-blockers, and *β*2-adrenergic agonists.

Blood pressure was measured using a sphygmomanometer adjusted to the dimensions of the patient's arm. Measurements of the blood pressure and pulse were taken after 5 minutes of dorsal decubitus, and at 1, 2, and 3 minutes after standing, to be continued if hypotension persists within the first 3 minutes. A decrease by more than 20 mmHg of systolic blood pressure or more than 10 mmHg of diastolic blood pressure was considered to be a positive reading confirming orthostatic hypotension after the meal.

Overall, 26 patients underwent these tests within a 6-month period. In 13 patients the diagnosis of DS was confirmed, while it was revoked in the other 13 patients.

## 3. Results

We divided the patients into 3 groups according to the kind of surgery that they had underwent (LSG, LRYGB with gastrojejunal mechanical (stapler) anastomosis or LRYGB with gastrojejunal manual anastomosis). Patients who underwent LSG (Group A) were 268 patients from 5 centers: 187 (69.7%) patients from CHRU Montpellier, 15 (5.6%) patients from CHU Nice, 32 (11.9%) patients from CHU Lille, 19 (7%) patients from CHU Nancy, and 15 (5.6%) patients from CHU Ambroise Paré. Patients who underwent LRYGB with a 30 mm linear mechanical stapler gastrojejunal anastomosis (Group B) were 229 patients from 3 centers: 112 (48.9%) from CHU Nancy, 77 (33.6%) from CHU Lille, and 40 (17.4%) patients from CHU Ambroise Paré. Patients who underwent LRYGB with a 15 mm manual anastomosis (Group C) were 44 (100%) patients from CHU de Nice.

The distinction between LRYGB patients with different gastrojejunal anastomosis was proposed due to the hypothesis of a relationship between the type and length of the gastrojejunal anastomosis and the risk of DS among these bariatric population.

The overall incidence of DS in these population was 8.5% at M1 and M6. We reported only 4 cases of DS (1.49%) at M1 and 3 cases (1.12%) at M6 in the LSG group. We also reported DS in 41 (17.9%) patients at M1 and in 43 (18.78%) at M6 in Group B. DS was present only in one patient (2.27%) at M1 and was absent (0%) at M6 in Group C.

Baseline data of the population under study is representative of typical bariatric surgery demographics with a high female-to-male ratio (75.6% of the patients were female), a mean age of 41 years, and a mean BMI of 45 kg/m^2^.

One month after surgery mean BMI dropped to 40.55 kg/m^2^, and at 6 months to 34.74 kg/m^2^.

### 3.1. Complications

There were no deaths. Global complication rate was 22.37% (121/541 patients).

Major complications were classified as those requiring surgical, endoscopic, or radiological intervention (score ≥ 3 according to the Clavien-Dindo classification [7]).

Fifty-five (20.5%) patients presented complications in Group A, although only 9 (3.36%) were considered major complications.  Table 2 summarizes the complications other than DS in the LSG group.

Sixty-six (24.18%) patients developed complications in LRYGB group (B + C). Major complications occurred in 15 (5.49%) patients.  Table 3 summarizes the complications other than DS among LRYGB patients.

## 4. Discussion 

The emergence of a bariatric surgery as an independent field of general surgery necessitates a careful and meticulous study of all possible complications, including DS, a long-term known possible consequence for patients undergoing gastrointestinal surgery.

As previously mentioned, symptoms of DS can be divided into early and late dumping with GI or vasomotor symptoms.

A number of peptides and vasoactive substances contribute to the pathogenesis of both types of dumping: neurotensin, vasoactive intestinal peptide, catecholamines, serotonin, and substance P [8].

The diagnosis of dumping syndrome is mostly clinical and relies on thorough history taking and answering questionnaires such as the Sigstad questionnaire and the Dumping Symptom Rating Scale, among others [4, 9]. Oral glucose provocation tests are useful when the diagnosis is doubted. Symptoms of early dumping can be elicited by an oral glucose challenge [10]. A rise in heart rate by 10 beats per minute or more in the first hour after an oral glucose challenge (with 50 g of glucose), following 10-hour fasting, is diagnostic. This test was found to be 100% sensitive and 92% specific [10]. A diagnosis of late dumping can often be confirmed by frequent blood sampling after provocation with oral glucose. In response to this test, elevated plasma levels of glucose during the first 60 minutes and reduced plasma glucose levels 1-2 hours later are expected. However, induction of symptoms after glucose provocation is more accurate for diagnosis of late dumping. Scintigraphic gastric emptying tests may assist in the diagnosis of dumping, especially in nonsurgical dumping, like in diabetes mellitus [11].

Dietary modifications are the mainstay of therapy in dumping syndrome. Dietary treatment of dumping focuses on the reduction of intake of simple carbohydrates. Fluid intake during meals should be restricted. Patients should be instructed to avoid liquids for at least 30 minutes after a solid meal. Milk and dairy products are not well tolerated and they should be avoided [11]. Daily food intake should be divided into at least six meals. Most patients with mild symptoms respond well to dietary changes. In patients with persisting symptoms, octreotide 25–50 g s.c. 30 min before meal has been shown to retard gastric emptying, slow small bowel transit, and inhibit release of vasoactive peptides [8].

DS incidence and severity have been correlated to the type of gastrectomy performed, suggesting a higher incidence of DS for patients undergoing total gastrectomy compared to proximal gastrectomy for early upper-third gastric cancer [2]. Currently, it has been proven that DS occurs mainly after LRYGB operation, although the symptoms tend to improve over time [3], and that DS may occur after procedures involving at least partial gastric resection or bypass, including LRYGB and LSG. Although restrictive interventions like LSG would be expected to carry a low risk of DS, two prospective studies reported that up to 40% of patients had symptoms suggestive of dumping syndrome 6–12 months after sleeve gastrectomy [12, 13].

The use of surgical methods for treating dumping syndrome is uncommon, even after the medical treatment has failed. Some authors have described laparoscopic reversal to normal anatomy with or without concomitant sleeve gastrectomy, after Roux-en-Y gastric bypass, as definitive method of treatment for intractable DS [13, 14].

In our study, we aimed to find the real incidence of DS among patients undergoing LSG and LRYGB, while differentiating between patients undergoing LRYGB with different gastrojejunostomy techniques, primarily 30 mm linear mechanical stapler gastrojejunal anastomosis and 15 mm manual gastrojejunal anastomosis. In the literature the correlation between the size of the anastomosis and occurrence or remission of the dumping syndrome was also demonstrated by Fernández-Esparrach et al. [15]. In this study endoscopic gastrojejunal anastomotic reduction was performed in 6 patients with intractable dumping syndrome after LRYGB using a combination of argon plasma coagulation, endoscopic suturing, and fibrin glue.

Patient demographics, short- and long-term weight loss results, and complication rate were similar to those reported in the literature.

When comparing DS incidence among patients of Group A and Group B, the bypass group with 30 mm mechanical anastomosis had a significant higher incidence of DS (*p* < 0.05). The hand sewn calibrated anastomosis was associated with a significantly lower incidence of DS if compared to group B (*p* < 0.05). The difference of incidence of DS between Groups A and C was not significant.

The relatively lower risk of DS in patients of Group C when compared with patients of Group B suggests a relationship between the type and length of the gastrojejunal anastomosis and the risk of DS in the population. This suggests that decreasing the length and performing a manual hand sewn gastrojejunal anastomosis may decrease the risk of DS in patients undergoing LRYGB.

Some of our patients had bariatric revisions. Most parts of them were conversions from band to LSG. These patients are more susceptible to developing surgical complications. Nevertheless we did not separate them in a different group. We believe DS incidence might not be increased among those patients even in complicated ones. This could be a question of debate.

## 5. Conclusion 

Although any procedure which involves gastrointestinal resection or digestive system bypass incurs the risk of developing DS, LSG is associated with a significantly lower risk of DS than LRYGB with 30 mm mechanical stapler anastomosis gastrojejunal anastomosis.

Patients undergoing LRYGB with 15 mm manual hand sewn gastrojejunal anastomosis may have a reduced risk of DS, which may suggest a possible relationship between the type and length of the gastrojejunal anastomosis.

The relatively short follow-up period of this study indicates the need for further analysis on a long-term basis.

## Figures and Tables

**Table 1:** Correlation of dumping symptoms following a meal within the Sigstad scoring system.

Shock	+5
Desire to lie or sit down	+4
Fainting, syncope, unconsciousness	+4
Breathlessness, dyspnea	+3
Palpitation	+3
Weakness, exhaustion	+3
Sleepiness, drowsiness, apathy, falling asleep	+3
Restlessness	+2
Dizziness	+2
Nausea	+1
Headaches	+1
Feeling of warmth, sweating, pallor, clammy skin	+1
Abdominal fullness, meteorism	+1
Borborygmus	+1
Eructation	−1
Vomiting	−4

**Table 2:** Complications other than DS after LSG.

Complication	Number of patients (*n*)	Treatment
Reflux +/− heartburn	21	Medical +/− endoscopy +/− pH 24 h esophageal pH monitoring
Vomiting	5	Diet modifications
Hemorrhage	7	4 surgical, 3 transfusion
Fistula	4	2 surgical, 1 prosthesis 1 surgical + endoscopic
Fistula + PE	1	Surgical
Abscess	3	ATB
Tachycardia	1	CT scan within normal findings
Dysphagia to meat	1	Diet modification
Dehydration	1	Rehospitalisation
LL sensory disorder	1	—
Cholelithiasis	1	Cholecystectomy
Failure to lose weight	2	—
Hypotension ± malaise ± hypoglycemia	2	Rest + NS IV infusion
Swelling of left internal jugular vein	1	—
Total	51	

**Table 3:** Complications other than DS after LGB.

Complication	Number of patients (*n*)	Treatment
Bleeding	2	Surgical
Cholelithiasis	1	Cholecystectomy
G-J anastomotic ulcer	2	PPI
Intra-abdominal abscess	4	Surgical
Internal hernia	1	Surgical
Anastomotic stenosis	5	Surgical/endoscopic
Perforation of the gastric remnant	1	Surgical
Vitamin B1 deficiency	2	Supplementation
Vitamin B12 deficiency	3	Supplementation
Fistula of G-J anastomosis	1	Surgical
Total	22	
